# MiR-let-7a inhibits cell proliferation, migration, and invasion by down-regulating PKM2 in cervical cancer

**DOI:** 10.18632/oncotarget.15999

**Published:** 2017-03-08

**Authors:** Man Guo, Xinying Zhao, Xiaolei Yuan, Jing Jiang, Peiling Li

**Affiliations:** ^1^ Department of Obstetrics and Gynecology, The Second Affiliated Hospital of Harbin Medical University, Harbin, Heilongjiang, P.R. China; ^2^ Department of Blood Dialysis, Heilongjiang Agricultural Reclamation Bureau General Hospital, Harbin, Heilongjiang, P.R. China

**Keywords:** miR-let-7a, PKM2, CC

## Abstract

In recent decades, miRNA has been reported as a crucial modulator in some biology progressions. This work aims to assess the expression and role of miR-let-7a and pyruvate kinase muscle isozyme M2 (PKM2) in CC tissues and cell lines. Here, we identified that miR-let-7a expression was decreased in CC tissues, and SiHa and HeLa cells (all *P* < 0.001), however, PKM2 expression was increased in these samples. Statistically, miR-let-7a was inversely associated with PKM2 mRNA or protein (*p* = 0.013, *p* = 0.015, respectively). *In-vitro* assays revealed that ectopic miR-let-7a expression repressed SiHa and HeLa cell proliferation, migration and invasion, and enhanced SiHa and HeLa cell apoptosis. Furthermore, luciferase reporter assays revealed the 3′-UTR of PKM2 was identified a target of miR-let-7a, by which miR-let-7a affected the expression of PKM2 in SiHa and HeLa cells. Besides, PKM2 plasmids partially abrogated the inhibitory effects of miR-let-7a, while si-PKM2 enhanced the inhibitory effects of miR-let-7a. *In vivo*, miR-let-7a mimics indeed repressed tumor growth in mice xenograft model. In conclusion, our results demonstrated that miR-let-7a inhibits cell proliferation, migration and invasion by down-regulation of PKM2 in cervical cancer. miR-let-7a/PKM2 pathway may be a useful therapeutic target for CC patients.

## INTRODUCTION

Cervical cancer is one of the most common gynecological cancers in the worldwide [1, 2]. Emerging evidence has indicated that human papillomavirus plays an important role in the initiation and progression of cervical cancer. It should be noted that only a part of patients were infected with HPV, while others have not HPV infection, suggesting that many cements were implicated in the progression and development of cervical cancer [3–5]. In spite of some related studies published recently, the etiology of cervical cancer is still largely unclear. Therefore, in this work, we endeavor to explore the signaling mechanisms that were involved in the development of cervical cancer.

MiRNAs are a kind of small, non-coding nucleotide RNAs, and interact with 3′-UTR of the related target mRNAs to regulate the expression of related genes by directly and indirectly affecting the transcription process of mRNAs. In addition, miRNAs also regulate the degradation of related target mRNAs [6, 7]. In recent years, miRNAs were reported to play an important role in some events involved in oncogenesis, such as cell survival, proliferation, apoptosis, migration and metastasis [8]. miR-let-7a acts as a kind of new miRNAs, and has been featured by a tumor suppressor in different human tumors. miR-let-7a also targeted some related genes to affect signal pathways of tumors. As reported miR-let-7a has a close relationship with PKM2 signal pathway, and miR-let-7a exerts an anti-tumor effect on different tumors [9–11]. However, the role of miR-let-7a in proliferation and invasion of cervical cancer remains unknown.

In the present study, we introduced the *in-vitro* assays to explore the expression model and the molecular mechanisms of miR-let-7a in the development and progression of cervical cancers. Our results demonstrated that the expression of miR-let-7a was obviously decreased in cervical cancer cells and tissues, and then down-regulation of miR-let-7a promoted cervical cancer cell proliferation by directly binding to 3′-UTRs of PKM2.

## RESULTS

### MiR-let-7a is decreased in CC tissues, SiHa and HeLa cell lines

**Figure 1:**
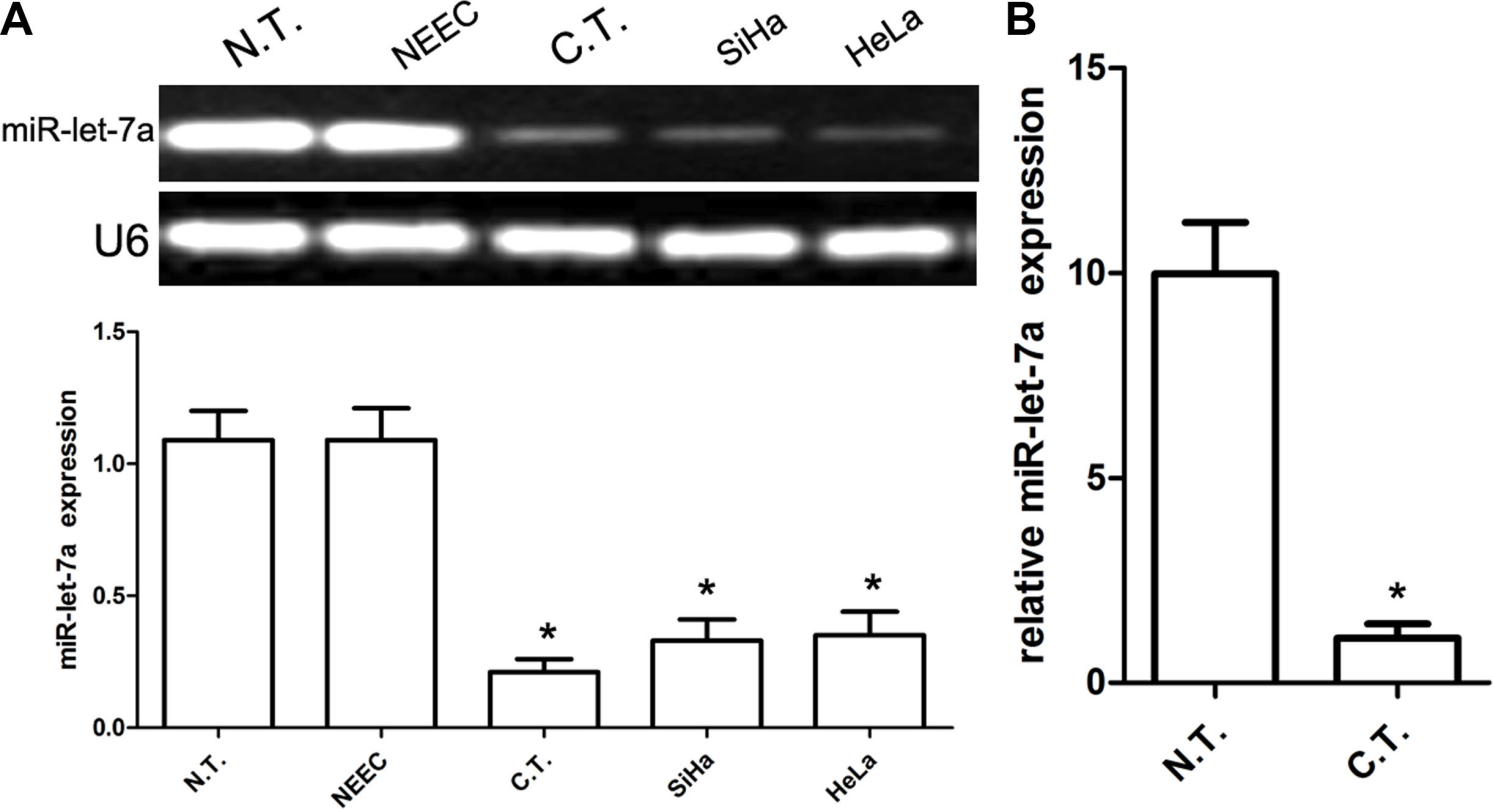
Expression of miR-let-7a in human CC tissues and cell lines (**A**) Relative miR-let-7a messenger RNA-expression levels in representative three samples and cell lines, including SiHa and HeLa were detected by RT-PCR analysis. The average expression was normalized to U6 expression. Each bar represents the mean of three independent experiments. (**B**) Real-time PCR analysis of miR-let-7a expression in 35 cases of cancer and paired normal tissues. The average expression was normalized to U6 expression. All data are represented as mean ± SEM of at least three replicate experiments unless otherwise noted. *denotes significance at *P* < 0.001 relative to normal cervical cancer tissues or NEEC by student *t*-test.

At first, we explored the expression of miR-let-7a in 35 pairs of CC tissues using quantitative RT-PCR analysis, and found that the expression of miR-let-7a was significantly decreased in cancer tissues compared with normal tissues. Subsequently, semi-quantitative RT-PCR analysis revealed that the expression of miR-let-7a was indeed decreased in cancer tissues compared with normal tissues. Consistent with tissues, SiHa and HeLa cell lines also revealed obviously down-regulated miR-let-7a expression as compared with human normal NEEC cells (*P* < 0.001) (Figure 1). In general, our findings indicated that miR-let-7a is decreased in CC tissues, SiHa and HeLa cell lines.

### PKM2 is elevated in CC tissues, SiHa and HeLa cell lines

**Figure 2:**
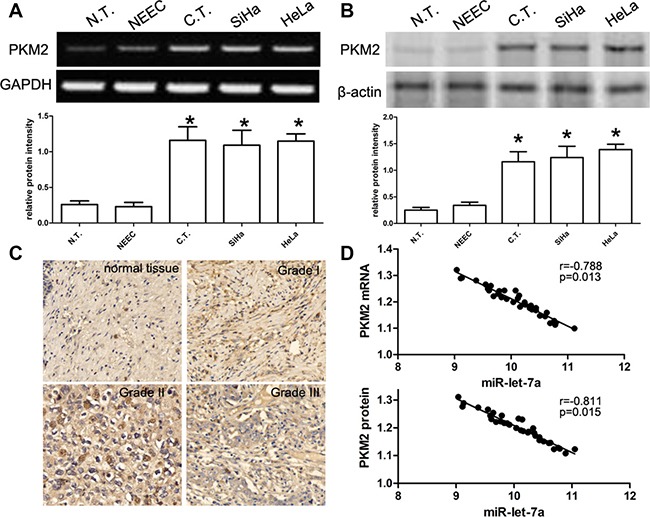
Expression of PKM2 in CC tissues and cell lines, and its correlation with the expression miR-let-7a (**A**) RT-PCR analysis of PKM2 mRNA expression in 35 cases of cancer tissues (C.T.), paired normal tissues (N.T.), and cell lines including SiHa and HeLa. Quantification analysis was defined as the relative density of PKM2 mRNA to GAPDH. GAPDH was used as an internal control. Results shown are the mean ± SEM of repeated independent experiments. (**B**) The expression of PKM2 protein was examined in 35 cases of CC tissues, paired normal tissues, and cell lines including SiHa and HeLa using western blot. The average PKM2 expression was normalized to β-actin expression. All data are represented as mean ± SEM of at least three replicate experiments unless otherwise noted. *denotes significance at *P* < 0.001 relative to normal cervical cancer tissues or NEEC by student *t*-test. (**C**) Representative immunostaining of PKM2 in non-neoplastic human cervical tissues and cervical cancer tissues, including cervical cancer grade 1, grade 2 and grade 3. (**D**) According to Pearson′s correlation analysis, the expression of PKM2 mRNA or protein in 35 cases of cervical cancer tissues was inversely associated with the expression of miR-let-7a (*p* = 0.013, *p* = 0.015, respectively).

According to previous reports, PKM2 expression is associated with miR-let-7a, thus we explored and analyzed the expression of PKM2 in CC tissues and cell lines using RT-PCR, qRT-PCR and western blot. We demonstrated that the expression PKM2 mRNA was significantly elevated in the cancer tissues when compared with that in normal squamous epithelial tissues. Consistent with tissues, SiHa and HeLa cell lines also revealed that the expression PKM2 mRNA was significantly elevated in when compared with human normal NEEC cells (*P* < 0.001) (Figure 2A). Western blot analysis further identified that the expression of PKM2 protein was obviously elevated in the cancer tissues, SiHa and HeLa cell lines (*P* < 0.001) (Figure 2B). Besides, we detected the expression of PKM2 protein in 35 cases of cervical cancer and their adjacent non-tumor cervical samples using IHC technology. Representative images of PKM2 protein expression were presented in Figure 2C. PKM2 was observed to be highly expressed in cancer tissues as compared with adjacent non-tumor samples. At last, we collected related data and identified that miR-let-7a was negatively associated with PKM2 mRNA or protein (*r* = −0.788, *p* = 0.013; *r* = −0.811, *p* = 0.015) (Figure 2D). Our findings suggested that PKM2 is elevated in CC tissues, SiHa and HeLa cell lines.

### miR-let-7a and PKM2 are correlated with clinical characteristics of cervical cancer

We also analyzed the relationship between miR-let-7a and clinical pathological characteristics of cervical cancer, 35 cases of CC tissues were separated into two groups (low miR-let-7a expression group and high miR-let-7a expression group) based on the median of relative intensity of miR-let-7a expression in cervical cancer tissues. The clinicopathological characteristics of 35 cervical cancer patients were shown in Table 1. We determined that low miR-let-7a expression in CC was obviously associated with advanced FIGO stage, lymph node metastasis and tumor size (all *P* < 0.000). However, the expression of miR-let-7a was not related to age (Table 1). Conversely, we observed that high PKM2 expression in cervical cancer tissues was obviously related to advanced FIGO stage, lymph node metastasis and tumor size (all *P* < 0.000). However, PKM2 expression was also not related to age (Table 1).

**Table 1:** Correlations of miR-let-7a and PKM2 with clinicopathological indicators of cervical cancer

Indicators	*N*	miR-let-7a	*p* value	PKM2 (OD values)	*p* value
		Expression level		Expression level	
Age					
< 45	15	2.2 ± 0.2	0.158	11.6 ± 2.9	0.563
≥ 45	20	1.9 ± 1.4		11.3 ± 1.9	
Tumor size					
< 5 cm	17	2.3 ± 0.5	0.001	13.7 ± 1.4	0.000
≥ 5 cm	18	1.7 ± 0.7		8.8 ± 1.1	
Grade					
I + II	16	2.5 ± 0.6	0.000	7.9 ± 1.2	0.000
III	19	1.5 ± 0.8		13.3 ± 1.7	
LN metastasis					
Yes	17	1.4 ± 0.5	0.000	13.6 ± 1.4	0.000
No	18	2.6 ± 0.7		8.5 ± 1.1	
FIGO stage					
I–II	18	2.5 ± 0.5	0.000	8.7 ± 1.8	0.038
III–IV	17	1.6 ± 0.7		14.2 ± 1.4	

### MiR-let-7a inhibits SiHa and HeLa cell proliferation

**Figure 3:**
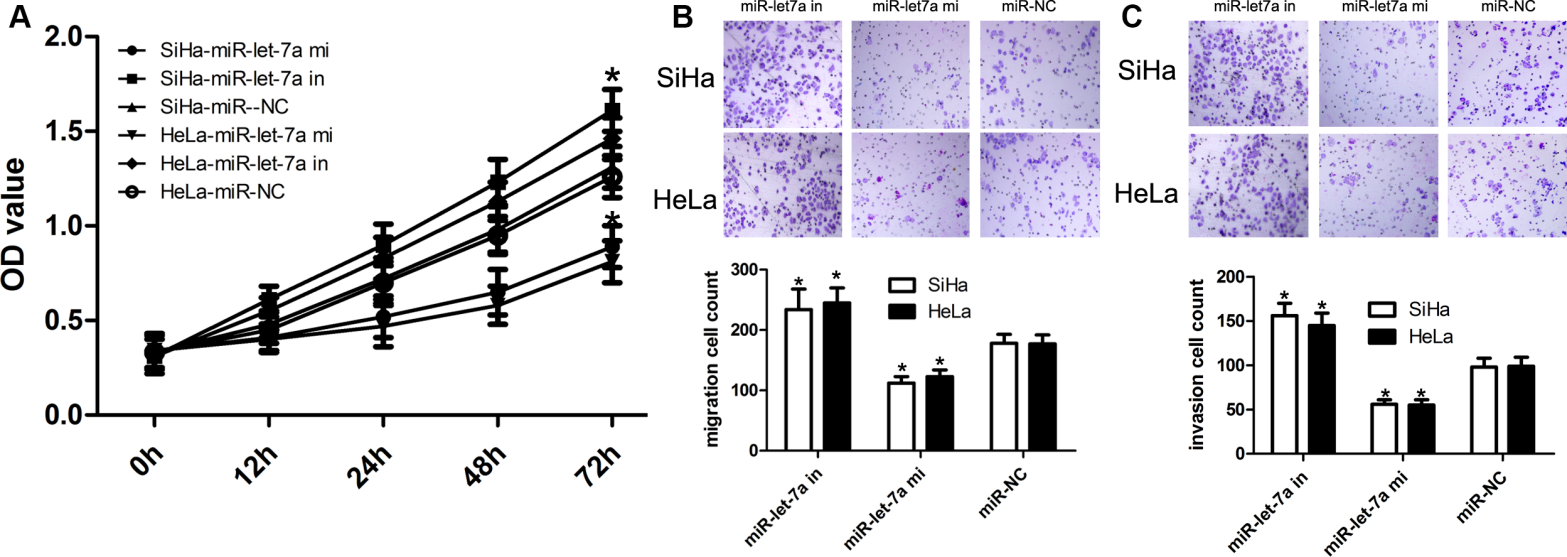
miR-let-7a upregulation inhibited SiHa and HeLa cell proliferation, migration and invasion (**A**) Validation of miR-let-7a expression levels after transfection with miR-let-7a mimics, miR-let-7a inhibitor, or the relative controls by RT-PCR analysis. CCK-8 assays revealed that up-regulation of miR-let-7a inhibited cell proliferation of SiHa and HeLa cells. *denotes significance at *P* < 0.01 relative to NC miRNAs by ANOVA. (**B**–**C**) Transwell assay was conducted to identify the role of miR-let-7a in migration and invasion, and up-regulation of miR-let-7a inhibited SiHa and HeLa cell migration and invasion. Representative micrographs and quantification of crystal violet-stained cell colonies were > 0.1 mm. Cells that penetrated the membrane were photographed at 100× magnification. Each bar represents the mean of three independent experiments. *denotes significance at *P* < 0.01 relative to NC miRNAs by student *t*-test.

In order to figure out the effect of miR-let-7a on cell proliferation, SiHa and HeLa cells were transfected with miR-let-7a mimics, miR-let-7a inhibitor, or their corresponding controls. We used qRT-PCR to identify the expression of miR-let-7a. Our findings showed that miR-let-7a mimics effectively inhibited the proliferation of SiHa and HeLa cells at 48 hours after transfection (Figure 3A). On the other hand, miR-let-7a inhibitor effectively promotes the proliferation of SiHa and HeLa cells at 48 hours after transfection (Figure 3A). Overall, these data showed that miR-let-7a inhibits SiHa and HeLa cell proliferation.

### MiR-let-7a inhibits SiHa and HeLa cell migration and invasion

In order to figure out the effect of miR-let-7a on cell invasion, SiHa and HeLa cells were transfected with miR-let-7a mimics, miR-let-7a inhibitor, or their corresponding controls. We used transwell assay to identify the role of miR-let-7a. Firstly, our findings showed that miR-let-7a mimics effectively inhibited the migration and invasion of SiHa and HeLa cells at 48 hours after transfection (Figure 3B, 3C). On the other hand, miR-let-7a inhibitor effectively promotes the migration and invasion of SiHa and HeLa cells at 48 hours after transfection (Figure 3B, 3C). Overall, these data showed that miR-let-7a inhibits SiHa and HeLa cell migration and invasion.

### MiR-let-7a facilitates SiHa and HeLa cell apoptosis

**Figure 4:**
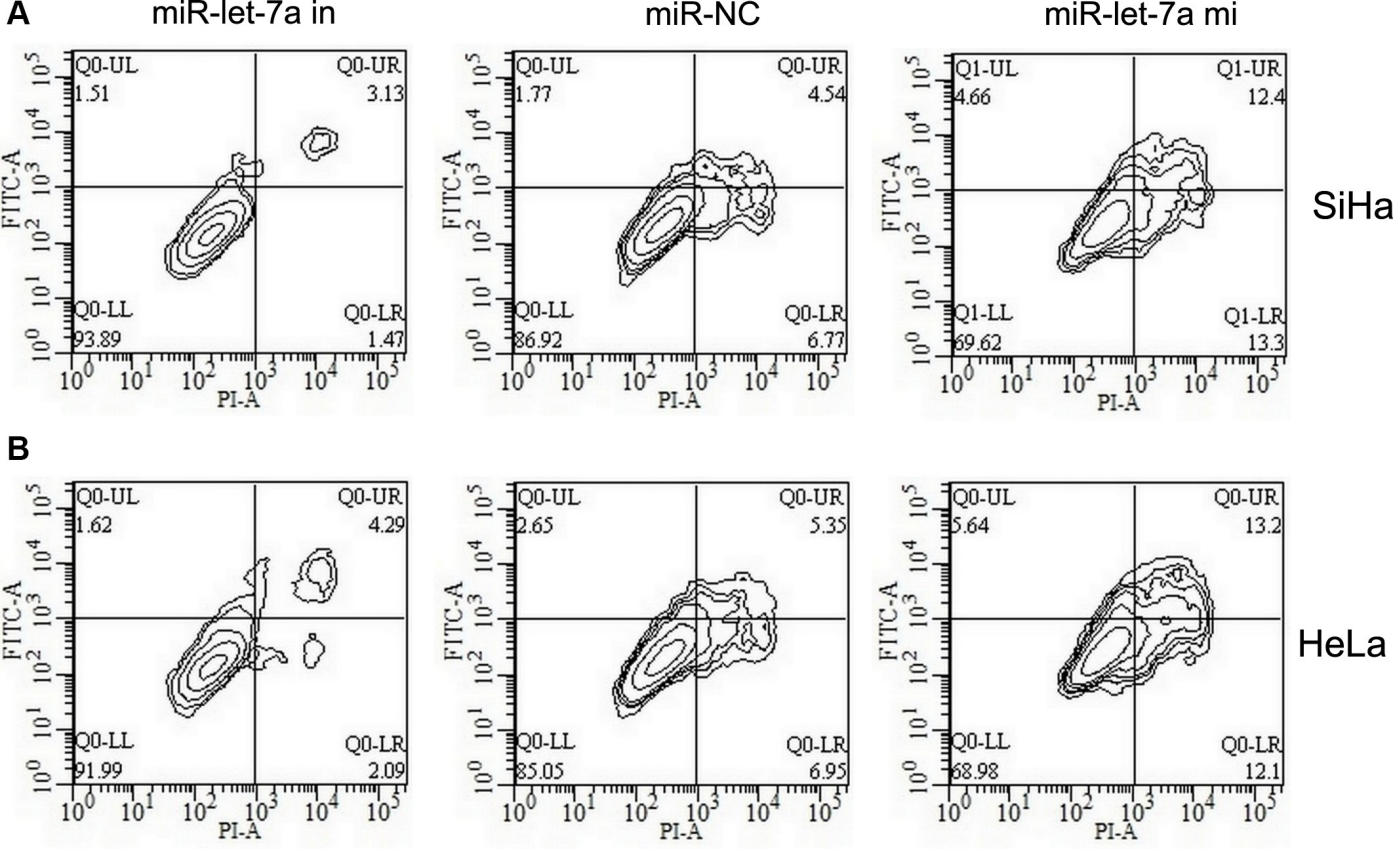
Effects of miR-let-7a on cell apoptosis in SiHa and HeLa cells SiHa (**A**) and HeLa (**B**) cells were transfected with miR-let-7a mimics, miR-let-7a inhibitor, or the relative controls as indicated for 48 h, and then cells were stained with Annexin V-FITC/PI, and analyzed by flow cytometry as described in methods. The statistic data were presented as mean ± SEM from three independent experiments. Representative images for cell apoptosis stained with Annexin V-FITC/PI.

With the results above, we used flow cytometry to conduct cell apoptosis assay. Here, SiHa and HeLa cells were transfected with miR-let-7a mimics, miR-let-7a inhibitor, or their corresponding controls. Our findings revealed that the apoptotic rate of SiHa and HeLa cells transfected with miR-let-7a mimics was 25.7% and 25.3%, respectively; while the apoptotic rate of SiHa and HeLa cells transfected with miR-NC was 11.3% and 12.3%, respectively. In addition, the apoptotic rate of SiHa and HeLa cells transfected with miR-let-7a inhibitor was 4.6% and 6.3%, respectively (Figure 4A–4B). The findings suggested that miR-let-7a facilitates SiHa and HeLa cell apoptosis.

### 3′-UTR of PKM2 is a direct target of miR-let-7a

**Figure 5:**
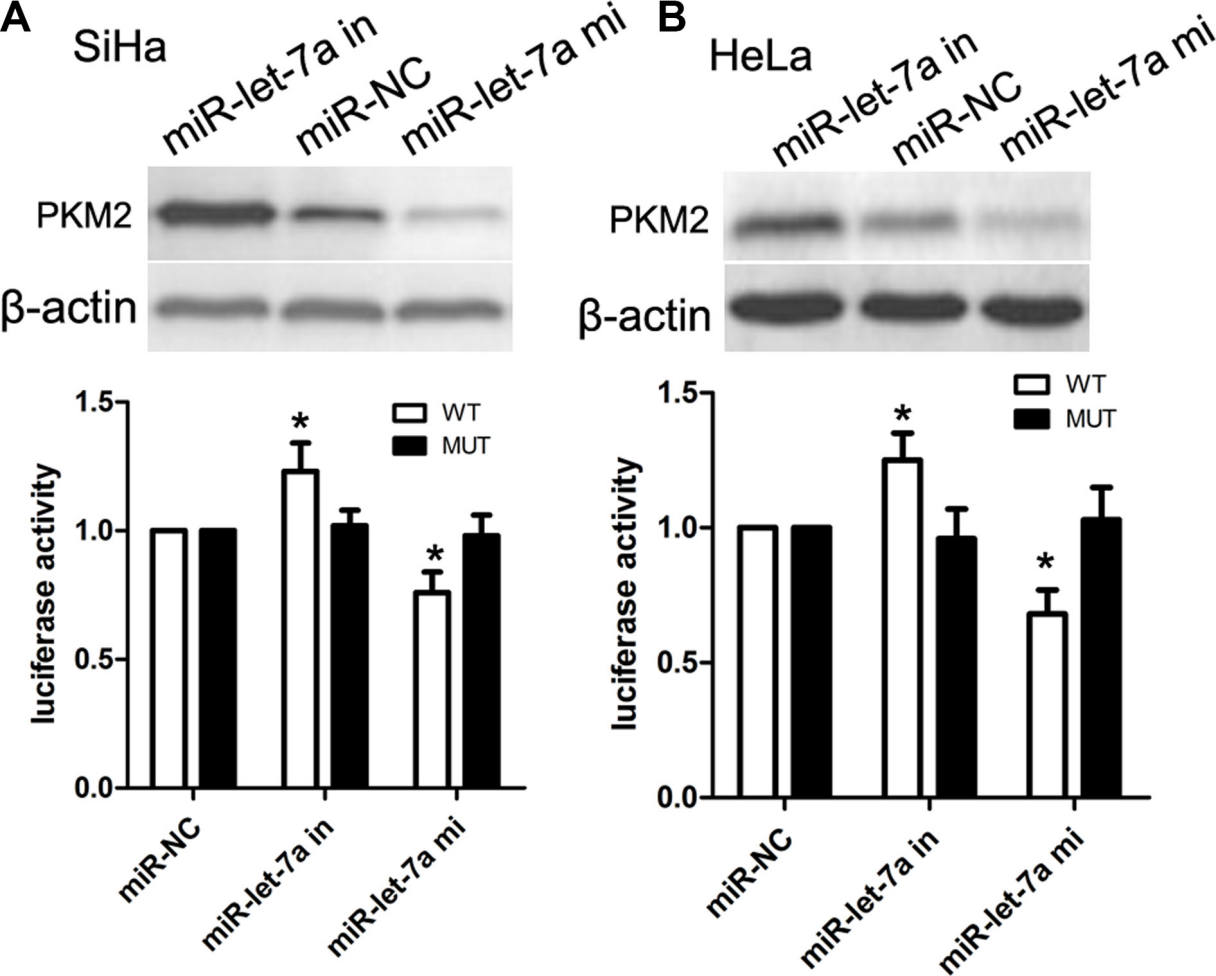
miR-let-7a suppressed PKM2 expression by directly targeting the PKM2 3′-UTR and altered levels of proteins related to proliferation in SiHa and HeLa cells (**A**) PKM2 protein expression in SiHa cells transfected with miR-let-7a or the miR-let-7a inhibitor was detected by Western blotting analysis. β-actin served as the loading control. Luciferase reporter assay of SiHa cells with the pGL3-PKM2-3′-UTR-wt or pGL3-PKM2-3′-UTR-mut were co-transfected with and miR-let-7a mimics, miR-let-7a-in or NC with increasing amounts (50 nM) of oligonucleotides. β-actin served as the loading control. *denotes significance at *P* < 0.01 relative to NC miRNAs by student *t*-test. (**B**) PKM2 protein expression in HeLa cells transfected with miR-let-7a or the miR-let-7a inhibitor was detected by Western blotting analysis. β-actin served as the loading control. Luciferase reporter assay of HeLa cells with the pGL3-PKM2-3′-UTR-wt or pGL3-PKM2-3′-UTR-mut were co-transfected with and miR-let-7a mimics, miR-let-7a-in or NC with increasing amounts (50 nM) of oligonucleotides. β-actin served as the loading control. *denotes significance at *P* < 0.01 relative to NC miRNAs by student *t*-test.

To describe the specific mechanism underlying miR-let-7a-modulated proliferation and apoptosis of cervical cancer cells, we utilized putative open-target prediction sources to explore potential targets of miR-let-7a, by which we selected PKM2 as a target because PKM2 plays an important role in regulating proliferation and apoptosis-related genes. In the present study, SiHa and HeLa cells were transfected with miR-let-7a mimics, miR-let-7a-inhibitor, or their respective controls. Afterwards, cells were co-transfected with the wild or mutant 3′-UTR vector of PKM2. Western blot analysis demonstrated that the expression of PKM2 protein was obviously decreased in cells tranfected with miR-let-7a mimics, while the expression of PKM2 protein was obviously increased in cells tranfected with miR-let-7a inhibitor (Figure 5A–5B). Dual-luciferase reporter assay identified that miR-let-7a mimics-transfected SiHa and HeLa cells revealed about 25% and 29% reduction in luciferase activity was observed respectively, whereas miR-let-7a-inhibitor promoted luciferase activity of wild-type PKM2. At the same time, when cells were co-transfected with miR-let-7a (mimics, inhibitor, or NC) and PKM2 3′-UTR-mut vector, the luciferase activity were not affected (Figure 5A–5B). Our findings indicated that PKM2 was a bona fide target of miR-let-7a.

### Over-expression of PKM2 abrogates miR-let-7a-induced inhibitory effects on cervical cancer

**Figure 6:**
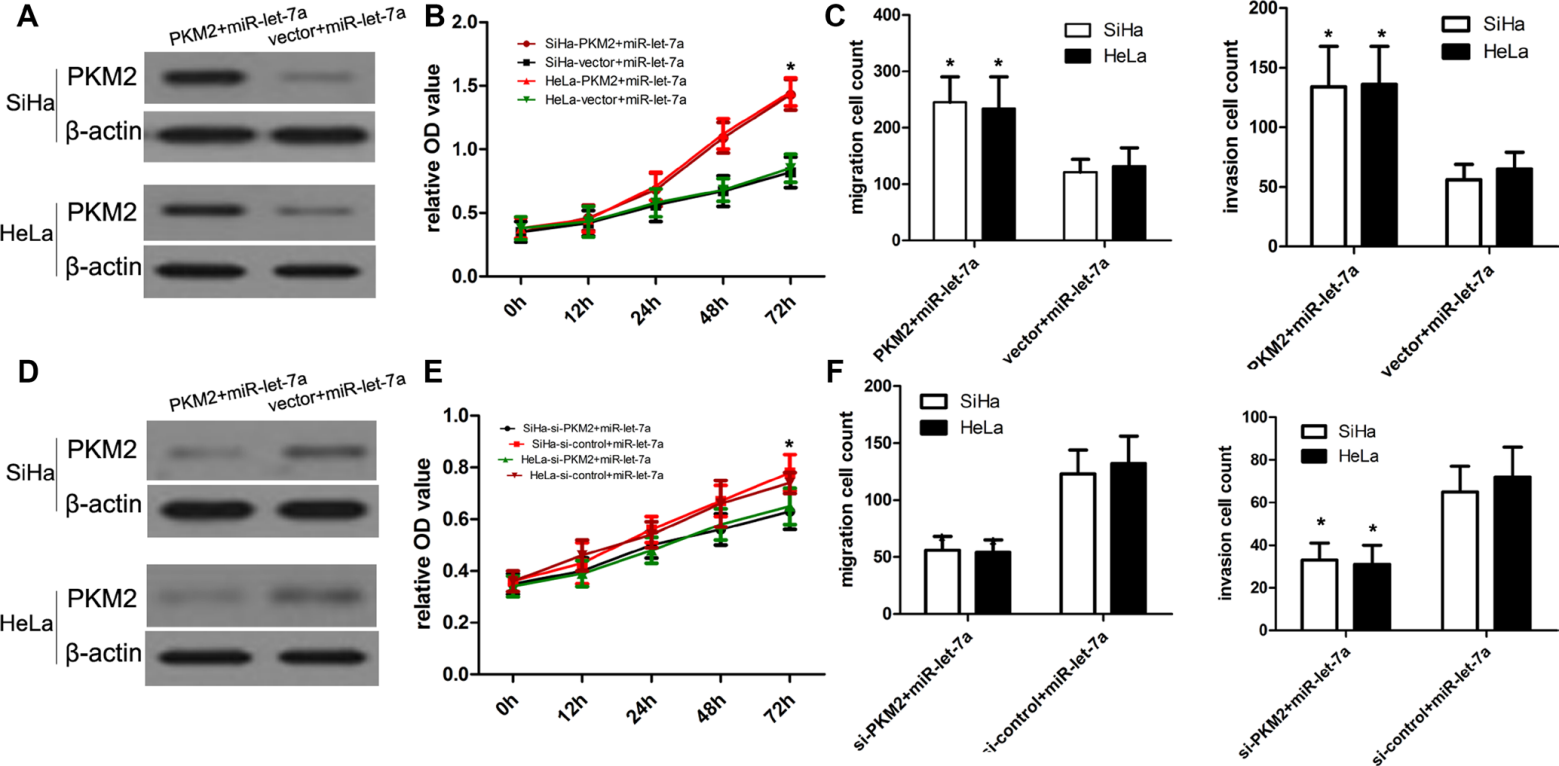
Effects of overexpression or inhibition of PKM2 on miR-let-7a-inhibited cell proliferation, migration and invasion (**A**) The proliferation capacity of miR-let-7a-overexpressing SiHa and HeLa cells was partially improved when cells were transfected with PKM2 plasmids in comparison with miR-NC. (**B**, **C**) The migration and invasion of miR-let-7a-overexpressing SiHa and HeLa cells were effectively improved when cells were transfected with PKM2 plasmids. *P < 0.001, vs. vector. (**D**) The proliferation capacity of miR-let-7a-overexpressing SiHa and HeLa cells was partially inhibited when cells were transfected with si-PKM2 compared with si-control. (**E**, **F**) The migration and invasion of miR-let-7a-overexpressing SiHa and HeLa cells were effectively improved when cells were transfected with si-PKM2. *P < 0.001, vs. si-control.

To elucidate the associations between miR-let-7a and PKM2, we transfected pcDNA3.1(+)-PKM2 plasmids into SiHa and HeLa cells with miR-let-7a mimics to over-express PKM2 protein (Figure 6A). The CCK-8 proliferation assay showed that enforced PKM2 expression promoted cell proliferation of SiHa and HeLa cells as compared with vector control (Figure 6B). Additionally, our transwell assay further identified that enforced PKM2 expression in SiHa and HeLa cells with miR-let-7a mimics promoted cell migration and invasion as compared with vector control (Figure 6C).

### Inhibition of PKM2 expression enforces miR-let-7a-induced inhibitory effects on cervical cancer

To elucidate the associations between miR-let-7a and PKM2, we transfected PKM2 siRNA and control siRNA into SiHa and HeLa cells with miR-let-7a mimics to deplete the expression of PKM2 protein. (Figure 6D). The CCK-8 proliferation assay showed that inhibited PKM2 expression affected cell proliferation of SiHa and HeLa cells as compared with si-control (Figure 6E). Additionally, our transwell assay further identified that inhibited PKM2 expression in SiHa and HeLa cells with miR-let-7a mimics affected cell migration and invasion as compared with si-control (Figure 6F).

### MiR-let-7a affects the growth of engrafted tumor

**Figure 7:**
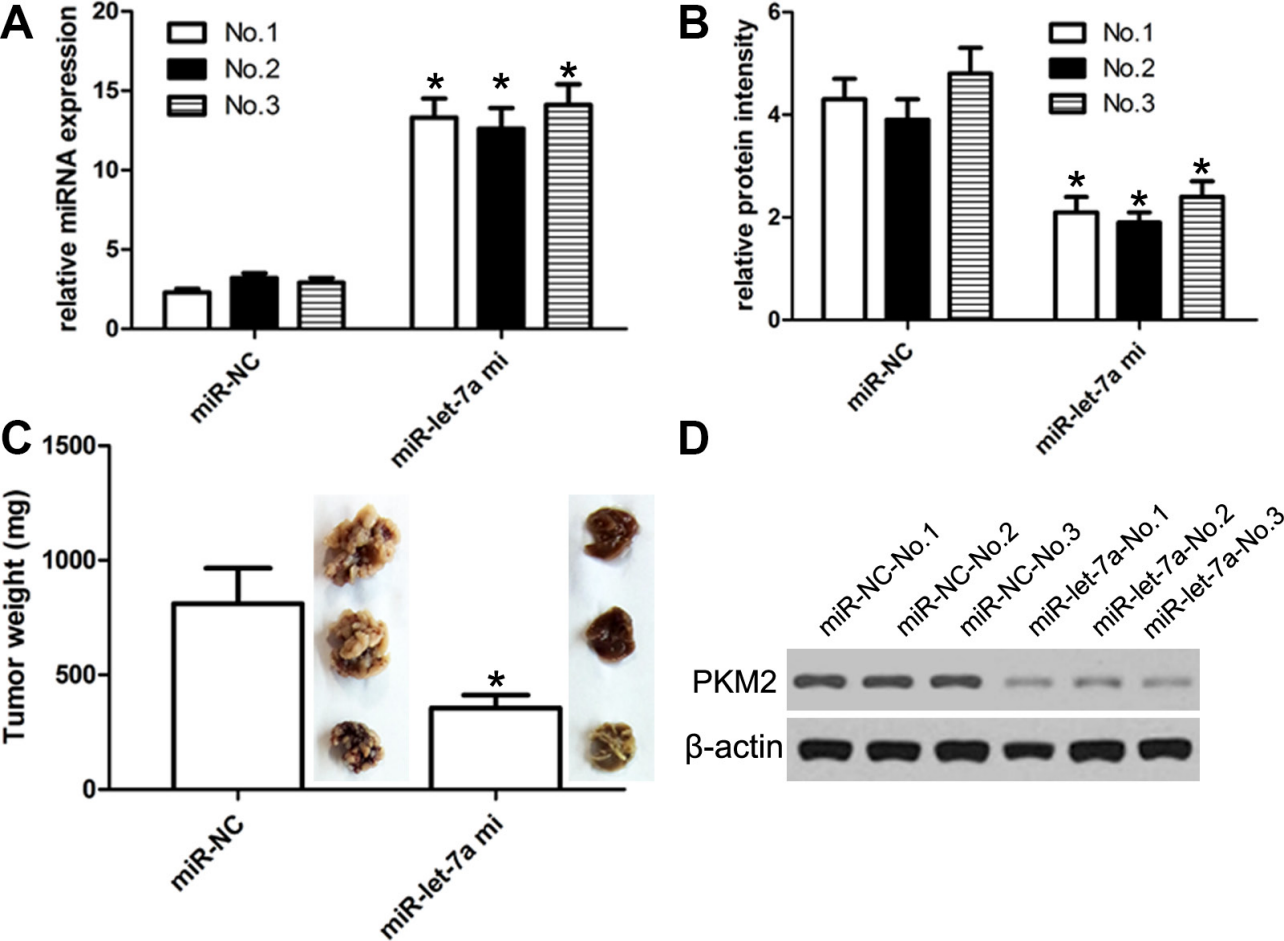
miR-let-7a affected the growth of SiHa-engrafted tumor SiHa cells stably transfected with miR-let-7a-expressing plasmids or empty vector were subcutaneously injected into nude mice (3 nude mice for experiment group, and 3 nude mice for control group) and tumor volumes were measured every week. Tumors were resected and weighed at 4 weeks after cell injection. Each injection contained 40 ng of miR-let-7a mimic in 10 μl saline solution. Mice were stitched and reanimated under warm light. Control mice were injected with mimic control. (**A**) The expression of miR-let-7a was detected using qRT-PCR assay. (**B**) The expression of PKM mRNA was detected using qRT-PCR assay. (**C**) We identified that miR-let-7a mimics could repress the SiHa-engrafted tumor growth when compared with control-induced SiHa-engrafted tumors. The weight of miR-let-7a-treated tumors was obviously lower compared with control-treated SiHa-engrafted solid tumor mass. (**D**) The expression of PKM2 protein detected using western blot. *P < 0.001, v.s. control.

Based on *in-vitro* assay, *in-vivo* tumorigenesis assay was carried out, and the experimental mice with intra-tumor injection of miR-let-7a survived normally as compared with control. We found no toxic effects of miR-let-7a on mice model. And then all xenograft tumors were excised, weighted and assayed. We identified that the expression of miR-let-7a was enhanced in SiHa-engrafted tumors with miR-let-7a mimics compared with control (Figure 7A). *in-vitro* analysis demonstrated that miR-let-7a mimics suppressed cell growth of SiHa-engrafted tumors with miR-let-7a mimics as compared with control because the weight value of SiHa-engrafted tumor with miR-let-7a mimics was obviously lower compared with that of control (Figure 7C). Besides, the expression of PKM2 became decreased in SiHa-engrafted tumors with miR-let-7a mimics compared with that in the control (Figure 7B, 7D).

## DISCUSSION

MiRNA acts as a kind of endogenous, small and noncoding RNAs, which controlled related gene expression depending on its binding to 3′-UTR regions of related mRNAs [12, 13]. More and more reports showed that miRNAs play an important role in regulating various biological behaviours, including cancer cell survival, apoptosis, oxygen stress, and maintenance of cell phenotype [6, 8, 10]. Altered expression of miRNAs is usually related to different kinds and types of tumors, sunch as breast cancer, gliomas, and liver cancer. Additionally, PKM2 is reported as a key rate-limiting enzyme in the process of glycolysis. It has been demonstrated that isoforms of PKM2 plays a key role in cancer cell metabolism, cell proliferation, migration, cell cycle, and maintenance of malignant phenotype. To date, the role and function of PKM2 in the development of cancers are still unclear [14, 15]. Ergo, PKM2 has become a useful and attracting target for therapeutic options. Previous reports suggested that the inhibition of expression of PKM2 affects cell proliferation and invasion of tumors, and induces cell apoptosis, and inhibits cell growth of tumors in animal models of tumors, including breast cancer, myeloma, prostate cancer, liver cancer, pancreatic cancer and lung cancer [16–19]. In addition, recent reports showed that down-regulation of PKM2 mediated by small interfering RNA markedly affected tumor cell growth and enhanced the sensitivity of tumor cells to some chemotherapy reagents, suggesting that PKM2 may be a potential target for the treatment of cervical cancer patients.

In the present work, the expression of miR-let-7a was obviously down-regulated in cervical cancer tissues as compared with matched adjacent normal samples. Consistent with tissue results, low expression of miR-let-7a was also found in SiHa and HeLa cell lines. In other tumors, the expression of miR-let-7a was also decreased in gastric cancer, liver cancer and renal cell carcinoma. Conversely, the expression of PKM2 mRNA and protein was also obviously up-regulated in cervical cancer tissues, as well as SiHa and HeLa cells. Statistically, we found that the expression of miR-let-7a was inversely related to that of PKM2 mRNA or protein, suggesting that the expression of miR-let-7a is associated with the expression of PKM2 in cervical cancer development.

Functionally, we further investigated the role and significance of miR-let-7a in cell proliferation and apoptosis. *In vitro*, over-expression of miR-let-7a obviously promoted cell growth of SiHa and HeLa cells. Colony-formation assay revealed that ectopic expression of miR-let-7a affected the colony-formation capacity of SiHa and HeLa cells. Till now, it has been demonstrated that miR-let-7a regulates some kinds of target proteins, and some target molecules were involved into cell apoptosis, and proliferation. At the same time, WEE1, CDK6, and Bcl-2 are reported and confirmed as a target of miR-let-7a, which are implicated into miR-let-7a-induced cell proliferation and apoptosis [9]. However, the molecular mechanisms of miR-let-7a action are not fully elucidated. Subsequently, we explored whether PKM2 was a direct target of miR-let-7a in SiHa and HeLa cells using dual-luciferase reporter assay.

Dual-luciferase reporter assay demonstrated that luciferase activity was significantly reduced in miR-let-7a-transfected SiHa and HeLa cells, respectively, while miR-let-7a inhibitor was able to increase luciferase activity of cells with wild-type PKM2 as compared with control. Notably, the luciferase activities of SiHa and HeLa cells co-transfected with miR-let-7a mimics and PKM2 3′-UTR-mut were not significantly affected. These findings suggested that PKM2 was a direct target of miR-let-7a. It has been reported that PKM2 can serve as direct targets of some miRNAs in some malignancies. For example, miR-122 specifically targets the 3′-UTRs of PKM2, resulting in the arrest of G0/G1 phase of cell cycle in liver cancer cells. Besides, over-expression of miR-122 obviously affects invasiveness of liver cancer cells [20]. In the present study, we offered a novel pathway of miR-122, by which miR-let-7a specifically binds to 3′-UTR regions of PKM2, and affected the cell proliferation of cervical cancer.

In conclusion, we demonstrated a novel and important role of miR-let-7a/PKM2 pathway in regulating proliferation, apoptosis and invasion in human cervical cancer. In addition to these findings, we also demonstrated that miR-let-7a binds the 3′-UTR of PKM2 mRNA to decrease the protein expression of PKM2. Our work suggested that restoration of miR-let-7a expression may be a potential therapeutic strategy in the treatment of cervical cancer patients.

## MATERIALS AND METHODS

### Ethics statement

Human tissue samples used in this study were obtained with written informed consent from the patients or their relatives. This study was approved by the Ethics Committee of The Second Affiliated Hospital of Harbin Medical University.

### Specimens

35 cases of cervical cancer and paired normal squamous epithelial tissues were obtained from operations performed from 2010 to 2012 in the department of gynecology of Second Affiliated Hospital of Harbin Medical University. The median patient age was 45 years (range: 19–68 years). Histopathological diagnoses were based on the WHO classification and clinical stages were in accordance with the International Federation of Gynecology and Obstetrics criteria. All the tumors were primary and the patient information and follow-up data were complete. No radio- or chemotherapy treatment was used in any patient before surgery.

### Immunohistochemistry (IHC)

Paraffin-embedded tissues were cut into 4 μm sections and rehydrated through a graded alcohol series, subjected to heating for antigen retrieval, allowed to cool to room temperature, immersed in 3% hydrogen peroxide for 5–10 min, and then rinsed with phosphate-buffered saline (PBS). The sections were incubated with normal goat serum to block nonspecific binding, followed by incubation overnight with primary antibodies at 4°C, and then rinsed with PBS. Subsequently, the sections were incubated with a secondary antibody for 15 min at 37°C, washed with PBS, and then stained with diaminobenzidine. Finally, the sections were counterstained with hematoxylin, dehydrated, and mounted.

### Cell culture

Normal human endocervical epithelial cell (NEEC) was generated from human endocervical samples obtained from biopsies of women (22–23 years of age) who underwent surgery for minor gynecological issues and had no underlying endocervical pathology. None of them had received hormonal therapy in the 3 months preceding sample collection. Samples were minced into fragments < 1 mm and subjected to mild collagenase digestion. NEEC was cultured to confluence in a steroid-depleted medium composed of 75% Dulbecco's Modified Eagle Medium and 25% MCDB-105 (Sigma, St. Louis, MO) supplemented with antibiotics, 10% human albumin and 5 mg/mL insulin (Sigma). Cell lines HeLa and SiHa cell line used in our study was obtained from the American Type Culture Collection. HeLa and SiHa cells were routinely cultured in DMEM (Invitrogen) supplemented with 5% fetal bovine serum (Sigma) at 37°C under 5% CO2.

### Isolation of total RNA and qRT-PCR

Total RNA were extracted from harvested cells using the Zymo Quick-RNA miniprep extraction kit (Zymo Research, CA, USA) according to the manufacturer's instructions. Quantitative reverse transcription PCR (qRT-PCR) was conducted. For the detection, 1 μg of total RNA per sample was converted to cDNA using the SuperScript VILO cDNA Synthesis Kit (Invitrogen, Carlsbad, CA, USA). cDNAs were amplified and detected using SYBR Green PCR Kit (Qiagen, Valencia, CA, USA). The GADPH was used as endogenous control for mRNA. For detection of the miRNA, the cDNA products were synthesized using miScript Reverse Transcription Kit (Qiagen, Valencia, CA, USA). The primers specific for miR-let-7a or endogenous control RNU6B were purchased from Qiagen. qRT-PCR was performed using miScript SYBR Green PCR Kit (Qiagen). All reactions were run in triplicate on Bio-Rad C1000 thermal cycler (CFX-96 real-time PCR detection systems, Bio-Rad). The fold change of miRNA or mRNA expression was calculated according to the 2−ΔΔct method. The primers used were as follows: for miR-let-7a forward: 5′-TGAGGTAGTAGGTTGTATAGTTAAA-3′, miR-let-7a reverse: 5′-AACGAGACGACGACAGACTTT-3′; PKM2 forward 5′-GTCGAAGCCCCATAGTGAAG-3′, PKM2 reverse 5′-GTGAATCAATGTCCAGGCGG-3′; and for GAPDH forward, 5′-CATCTTCCAGGAGCGAGA-3′ and GAPDH reverse 5′-TGTTGTCATACTTCTCA-3′ (Invitrogen, Shanghai, China).

### Western blot

Cells were lysed in RIPA buffer (1% NP-40, 0.5% sodium deoxycholate, 0.1% SDS in PBS). Complete protease inhibitor cocktail (Roche, Indianapolis, IN, USA) was added to lysis buffer before use. Protein concentration was determined by Bio-Rad DC protein assay (Bio-Rad, Hercules, CA, USA). 20 ug of total protein from cell lysate was subjected to SDS-PAGE and transferred to nitrocellulose membrane. The membrane was blocked in 5% non-fat milk in PBS overnight and incubated with primary antibody. After washing for 30 min, secondary goat anti-mouse IgG (Vector Co., Burlingame, CA, USA) was applied to nitrocellulose membrane in TBS-Tween for 1 h. After washing, the proteins of interest were detected using Chemiluminescent HRP Antibody Detection Kit (Denville Scientific, South Plainfield, NJ, USA). Anti-PKM2 polyclonal antibody was purchased from Sigma-Aldrich (St. Louis, MO, USA). Anti-β-actin antibodies were purchased from Cell signaling technology (Danvers, MA, USA). The protein signals were captured using an electrochemiluminescent system (PerkinElmer Life Science, Boston, MA, USA).

### miRNA mimic transfection

miR-let-7a mimic (Life technologies, Shanghai, China), and corresponding negative controls miR-NC (GenePharma, Shanghai, China) were transiently transfected into cells as reported previously [18]. Cells were seeded on a 24-well plate at 10,000 cells/well. After 14 hours, cells were transfected with a miRNA mimics at a final concentration of 10 nm using Lipofectamine 2000 (Invitrogen, Carlsbad, CA, USA) according to the manufacturer's instructions. At post-transfection 48 hours, cells were harvested for western blot or qRT-PCR analyses.

### Transfection of plasmids

We used Lipofectamine 2000 transfection reagent (Invitrogen, Carlsbad, CA) to carry out transfection of plasmids. In this work, PKM2 cDNA without carrying its 3′UTR was inserted into pcDNA3.1(+) vector to generate the recombinant pcDNA3.1(+)-PKM2 plasmid. At the same time, the pcDNA3.1(+) vector acts as control. All constructs were identified for sequence correctness using direct sequencing technology (Beijing Aodingsheng Corp., Beijing China).

### Luciferase reporter assay

A pmirGLO Dual-Luciferase miRNA Target Expression Vector was used for 3′UTR Luciferase assays (Promega, Madison, WI, USA). The target genes of miR-let-7a were selected based on target scan algorithms [microRNA.org (http://www.microrna.org/microrna/home.do) Microcosm (http://www.ebi.ac.uk/enright-srv/microcosm/htdocs/targets/) and TargetScan (http://www.targetscan.org/)]. The miR-let-7a mimics, negative control and miR-let-7a inhibitor were purchased from RiboBio (Guangzhou, Guangdong, China). For 3′UTR luciferase assay, cells were co-transfected with hsa-miR-let-7a mimics or pmirGLO Dual-Luciferase miRNA Target Expression Vectors and wild type or mutant target sequence using Lipofectamine 2000. Luciferase assay was performed using the Dual-Luciferase^®^ Reporter Assay System (Promega) after transfection at 48 h. Data are presented as the mean value ± SEM for triplicate experiments.

### Cell proliferation assay

Cell proliferation was evaluated using a Cell-Counting Kit 8 (CCK8), as described by the manufacturer (Dojindo Molecular Technologies, Inc., Kumamoto, Japan). Two thousands of DU145 or PC-3 cells per well were cultured in 96-well plates and 10 μL of CCK-8 solution was added to each well at the indicated time points after transfection. Cells were further incubated for 2 h at 37°C in a 5% CO2 incubator. The absorbance was measured at 450 nm with Multiscan FC Microplate Photometer (Thermo Fisher Scientific, Rochester, NY, USA).

### Transwell assays

As for transwell assays, cells were cultured in the upper chambers of Matrigel-coated wells (1:5 dilution in serum-free medium) (Corning Costar, Cambridge, MA, USA), and then we supplemented 10% serum into the lower chamber, and then cells were cultured. After 24 h, we removed all cells loafed on the upper chambers of Matrigel-coated wells, and then cells on the lower surface of the chamber were stained using 0.1% crystal violet (Sigma, St. Louis, MO), and then cells were counted.

### Cell apoptosis assay

Cells were treated as mentioned above. After 36 h, cells were harvested, washed and resuspended with binding buffer (Sungene, China). Cells were incubated with Annexin V-FITC for 20 min and propidium iodide (PI) for 5 min at room temperature in darkness before analyzing with flow cytometer.

### *In vivo* tumorigenesis assay

The experiments involving animals were performed in accordance with the institutional guidelines for animal care and were approved by the University Committee for the Use and Care of Animals. Male BALB/c nude mice (4 weeks old and weighing about 20 g) were purchased from Shanghai Laboratory Animal Center (Shanghai, China). miR-let-7a-overexpressing or control cells (5 × 106 per mouse, 3 mice per group) were subcutaneously injected into the right flanks of mice. Tumor size was measured every week using a caliper. At 28 days after cell injection, animals were sacrificed and xenograft tumors were excised and weighted. We detected the tumor length, width and weight every 3 days. All mice in this study were euthanized by way of cervical traction at 25 days following tumor cell inoculation.

### Statistical analysis

The results represent the mean ± standard deviation (SD). Differences in the data were tested for statistical significance using two-way ANOVA or one-way ANOVA. For all tests, *P* < 0.05 was considered to be statistically significant.
